# CLINICAL FACTORS ASSOCIATED WITH INCREASED SEDENTARY TIME IN VERY ACTIVE OLDER ADULTS

**DOI:** 10.1093/geroni/igz038.598

**Published:** 2019-11-08

**Authors:** Kenneth Madden, Jocelyn Chase

**Affiliations:** 1 Gerontology and Diabetes Laboratory, University of British Columbia, Vancouver, British Columbia, Canada; 2 Division of Geriatric Medicine, University of British Columbia, Vancouver, British Columbia, Canada

## Abstract

Sedentary behavior (such as sitting) has been shown to be an independent risk factor for increased frailty and less successive aging, even in active individuals. Our study examined the clinical factors most associated with higher sedentary times (ST) in very active older adults. We recruited 54 adults from a Master’s ski team (Whistler, British Columbia; mean age 71.5±0.6 years, 55% female). Activity levels were measured using an accelerometer (SenseWear) worn continuously for 7 days. ST was defined as a lack of activity when not in the supine position, in order to exclude time spent sleeping. Potential predictor variables consisted of metabolic syndrome criteria (blood pressure, high density lipoprotein, waist circumference, triglyceride levels, fasting blood glucose), age, biological sex and heart rate. Predictors associated with ST (p<0.10) were entered into a stepwise multivariate regression model. Our subjects were extremely active, engaging in moderate to vigorous physical activity for 2.6±0.2 hours per day, greatly exceeding current activity guidelines. Despite these high activity levels, they were also sedentary for an average of 9.4±0.2 hours per day. Our final minimum effective model showed that waist circumference had a significant association with ST (Standardized **β** = 0.36±0.13, p=0.007), explaining 18% of the variation in ST. People are often subjectively unaware of how long they spend sedentary. Our study suggests, that in addition to promoting leisure time physical activity, physicians should also objectively measure ST in highly active older patients with high waist circumferences.

